# The Role of Personality in Daily Food Allergy Experiences

**DOI:** 10.3389/fpsyg.2018.00029

**Published:** 2018-02-06

**Authors:** Tamlin S. Conner, Miranda Mirosa, Phil Bremer, Rana Peniamina

**Affiliations:** ^1^Department of Psychology, University of Otago, Dunedin, New Zealand; ^2^Department of Food Science, University of Otago, Dunedin, New Zealand

**Keywords:** food allergy, gluten, personality, stress, mood, health, openness to experience, openness

## Abstract

Food allergies present numerous challenges to coping in everyday life. Even simple things like planning a lunch with a friend can be stressful for people with food allergies. But are some people more adversely impacted by having a food allergy than other people? This paper addressed this question by investigating whether individual differences in the Big Five personality traits (neuroticism, extraversion, openness, agreeableness, and conscientiousness) are related to food allergy-related problems in everyday life among adults with food allergies. Participants were 108 adults (85% female; mean age = 40.2; age range 18–87) with a physician-diagnosed food allergy [most commonly to gluten (54.6%), peanuts (21.3%), cow's milk (16.7%), and shellfish/seafood (16.7%)]. Participants completed an initial online survey that measured demographics, food allergy information, and personality traits using the Big Five Inventory (John et al., 1991). For 2 weeks, participants completed a daily online survey that queried the occurrence of 25 food allergy issues that day and participants' overall stress and mood that day. Neuroticism did not predict more frequent allergy issues or greater stress/poorer mood on days with more allergy issues. Instead, higher openness to experience predicted a range of issues including going hungry because there is no safe food available, problems finding suitable foods when grocery shopping, feeling anxious at social occasions involving food, being excluded, and feeling embarrassed and poorly understood about their food allergy. Conscientious people were less embarrassed or self-conscious about their food allergy, but they had more problems eating out, and their positive mood was more impaired by allergy issues than their less conscientious peers. Extraversion and agreeableness played minor roles. Personality testing can identify people that may have difficulty living with food allergies–such as those higher in openness to experience.

## Introduction

Food allergies affect between 1% and 10% of the population (Sicherer, 2011; Reilly and Green, 2012; Nwaru et al., 2014; Acker et al., 2017). Using the electronic health records of 2.7 million patients in the United States, Acker et al. (2017) found that 3.6% of patients had a diagnosed food hypersensitivity, which was split roughly equally between people with a probable immunoglobulin E (IgE)-mediated allergy to foods like cow's milk, tree nuts, peanuts, and shellfish, and the other half presenting with non-IgE mediated food allergies (e.g., coeliac disease) or non-allergic food hypersensitivities/food intolerances. A meta-analysis of 42 studies by Nwaru et al. (2014) further estimated the prevalence in the general population of some common food allergies such as cow's milk 0.6%, tree nuts 0.5%, soy 0.3%, peanuts 0.2%, egg 0.2%, wheat 0.1%, fish 0.1%, and shellfish 0.1%. Coeliac disease has been found to affect around 1% of the general population in Canada (Jamnik et al., 2017), Europe (Mustalahti et al., 2010), and New Zealand (Cook et al., 2004).

Food allergies can significantly impact stress levels and impair quality of life (Flokstra-de Blok et al., 2010; Peniamina et al., 2014, 2016). Managing a food allergy involves dealing with innumerable dietary and lifestyle changes brought about by the need to avoid ingestion of the culprit food allergen/s. Individuals with food allergies face issues such as recurring physical symptoms because of accidental ingestion of the allergen/s, social discrimination/stigma, difficulties finding safe foods to eat, and having to constantly check food for safety, which influence their ability to cope (Peniamina et al., 2014, 2016). An understanding of the factors that contribute to successful illness adaptation can be useful to inform clinical practice as well as to improve theoretical knowledge of the links between psychological and physical factors in people with food allergies.

Given that personality shapes the perception, interpretation, and behavior of individuals in relation to their experiences (John et al., 2008), it is likely that personality modulates the food allergy experience. Published research has not assessed how personality might influence the frequency and types of food allergy issues that people experience or their daily psychological reactions to food allergy issues such as heighted stress. However, the Big Five personality traits have been linked to illness adaptation and to indicators of illness adaptation (e.g., quality of life, perceived physical health, and perceived psychological health) in individuals with other chronic conditions (e.g., asthma, irritable bowel syndrome, chronic obstructive pulmonary disease, cancer, congenital heart disease, and diabetes) (De Clercq et al., 2004; Van De Ven and Engels, 2011; Rassart et al., 2013, 2014; Muscatello et al., 2016; Topp et al., 2016).

The most consistent finding from these studies is that the personality trait characterized by high negative emotion (neuroticism) is associated with poorer illness adaptation and reduced quality of life. These patterns have been shown in adults with Type 1 diabetes (Rassart et al., 2014), adults with chronic obstructive pulmonary disease (Topp et al., 2016), adolescents with asthma (Van De Ven and Engels, 2011), and adolescents with congenital heart disease (Rassart et al., 2013). Neuroticism has also been associated with greater symptom severity in adult patients with irritable bowel syndrome (Muscatello et al., 2016) and in adolescent asthma patients (Van De Ven and Engels, 2011), and has been associated with over-reporting health symptoms more generally (Larsen, 1992).

Probably the second most consistent pattern is that the personality trait characterized by less cooperation (low agreeableness) predicts poorer illness adaptation and quality of life for some populations and chronic illnesses (e.g., Van De Ven and Engels, 2011; Rassart et al., 2013, 2014; Muscatello et al., 2016); however, this pattern is not always found. For example, one study found that *high* agreeableness was linked to poorer disease coping among patients with chronic obstructive pulmonary disease (Topp et al., 2016).

The patterns for the remaining personality traits are less consistent. The trait associated with poor follow-through (low conscientiousness) has been associated with poorer illness adaptation mainly in adult populations (Rassart et al., 2014; Muscatello et al., 2016; Topp et al., 2016), but not in adolescent populations (Van De Ven and Engels, 2011; Rassart et al., 2013). By contrast, the personality trait associated with positive emotions (extraversion) appears more relevant to adolescent populations, with evidence that lower extraversion was linked to poorer quality of life in adolescents with asthma and adolescents with congenital heart disease (Van De Ven and Engels, 2011; Rassart et al., 2013). The desire for novel ideas or experiences (openness) has not been related to poorer illness adaptation or reduced quality of life in these studies.

In summary, the results from studies of the personalities of people living with other chronic conditions indicate that personality traits modulate their illness experiences in various ways. However, it is also evident that the relationship between personality traits and illness adaptation can differ depending on the type of chronic illness and the population. Thus, while earlier research can inform theories about how personality traits may modulate the experiences of people living with food allergies, research is needed to determine the role of personality in food allergy-related experiences.

The current study tested in sample of adults living with food allergies: (i) the role of personality on the frequency and type of food allergy issues experienced in daily life; and (ii) whether personality moderated the naturalistic relationship between food allergy issues and daily feelings of stress and mood (i.e., whether people felt more stress and worse mood on days with more allergy issues). The current study uses the same 2-week daily diary dataset as Peniamina et al. (2016), but focuses on different hypotheses related to personality. Based on knowledge of the characteristics associated with different Big Five personality traits (neuroticism, extraversion, openness, agreeableness, and conscientiousness) and the role of personality in the experience of other chronic conditions, we made the following hypotheses.

First, we hypothesized that, similar to results reported for other chronic conditions, neuroticism would be associated with experiencing more frequent allergy issues in daily life, and could exacerbate the negative association between allergy issues and daily stress because neuroticism is associated with a disposition to experience greater stress reactivity and poorer coping (Bolger and Schilling, 1991; Suls and Martin, 2005; Van De Ven and Engels, 2011; Rassart et al., 2013, 2014; Kööts-Ausmees et al., 2016; Muscatello et al., 2016; Topp et al., 2016).

Second, we hypothesized that extraverts may cope better because of their tendency to experience more positive emotions and be more assertive (John et al., 2008), with research having previously linked extraversion to positive health behaviors and better perceived health (Jerram and Coleman, 1999; Kööts-Ausmees et al., 2016).

Third, we hypothesized that individuals high in openness to experience might face greater issues with food allergies because the requirements of managing a food allergy—such as being cautious, eating known foods, and minimizing food novelty—are in direct conflict with their personality (John et al., 2008; DeYoung, 2010). People higher in openness also have broader, more complex lives (John et al., 2008; DeYoung, 2010), which could expose them to more situations where food allergy issues might occur.

Fourth, similar to Topp et al. (2016) we hypothesized that agreeable people would actually experience *more* food allergy issues in daily life and possibly greater impact of the issues because dealing with food allergies can put them in disagreeable positions where they are having to assert their needs.

Fifth, we hypothesized that conscientious individuals may experience fewer issues and less impact from allergy issues on psychological functioning because trait-specific behaviors such as planning, organizing, and prioritizing will enhance their ability to manage their condition (John et al., 2008). Previous research has linked higher conscientiousness with better illness adaptation, a lower reported impact of illness, and higher self-rated health (Rassart et al., 2014; Kööts-Ausmees et al., 2016; Muscatello et al., 2016; Topp et al., 2016).

## Materials and methods

### Participant recruitment

Adults with food allergies were recruited across New Zealand by placing advertising flyers at prominent locations (e.g., doctor's clinic noticeboards, supermarkets, libraries, universities, hospitals), through social media (advertising on Facebook), and by newspaper advertising across the country. The study was advertised as a research study about the “daily experiences of people living with a food allergy.” There were three inclusion criteria: Participants needed to be at least 18 years old, live in New Zealand, and self-report a medically-diagnosed food allergy. For the purpose of this study, food allergy was defined as a reproducible adverse reaction caused by an immune-mediated response to a food or food component. This definition is based on the World Health Organization definition of food allergy, and includes both IgE-mediated food allergies (e.g., peanuts, cow's milk) and non-IgE-mediated food allergies (e.g., coeliac disease) (World Health Organisation International Food Safety Authorities Network, 2006). This study was approved by two departmental ethics committees (Departments of Food Science and Psychology).

### Procedure

Participants accessed the study from anywhere in New Zealand using a website. After answering several eligibility questions (“Are you 18 years of age or older?,” “Do you live in New Zealand?,” “Have you been diagnosed with a food allergy by a general practitioner (GP) or allergy specialist doctor?”), they were given more information about the study, and then prompted to electronically sign informed consent. Following this, participants completed an initial online survey prior to signing up for a 2-week period during which they would complete 14 consecutive daily surveys over the internet. A 2-week period (including 2 weekends) was used for the daily surveys to allow for the experience of a broad range of issues while keeping participant burden to a minimum. Participants were given options of several upcoming 2-week periods, all of which started on a Monday. They were then asked to complete the daily survey each day, which was accessible through a password protected website between 6 p.m. and 2 a.m each day during their chosen 2-week period. A reminder email was sent out on the day of the first daily survey, and text reminders were sent out each evening during the 2-week period. The data were collected between January and July 2013. The majority of surveys were completed between 8 p.m. and 10 p.m. Participants were entered in a prize draw (one prize of NZ$200 and eight prizes of NZ$100) as a thank you for their participation and were given the option to receive an individualized report of the study results.

### Measures

The initial online survey collected socio-demographic variables (age, gender, and ethnicity), information about the type of food allergy and common symptoms, and more detailed information about how their food allergy was diagnosed to confirm they met the inclusion criteria of having a medically-diagnosed food allergy (i.e., they were asked the type of doctor who made the diagnosis and method/s of diagnosis). This was followed by the 44-item Big Five Inventory (BFI-44) measure of personality (John et al., 1991, 2008). Participants rated 44 behavioral statements (I see myself as someone who… “Is talkative”; … “Is depressed, blue”) on a 5-point scale with the response options of 1 (Disagree strongly), 2 (Disagree a little), 3 (Neither agree nor disagree), 4 (Agree a little), or 5 (Agree strongly). Scores for the behavioral statements associated with each of the five traits (neuroticism, extraversion, openness, agreeableness, conscientiousness) were averaged, reverse scoring when required (Chronbach's alphas > 0.80).

The first page of the daily diary survey collected information about participants' overall mood and stress that day. First, participants were asked “How much does each of the following words describe how you felt TODAY?” The mood items were: happy, irritable, enthusiastic, sad, content, and anxious, in that order. Each item was answered on a 5-point scale [1 (Not at all), 2 (Slightly), 3 (Moderately), 4 (Very much), or 5 (Extremely)]. The three negative mood items (sad, anxious, irritable) and the three positive mood items (content, happy, enthusiastic) were selected to capture a range of low to high activation negative and positive mood states (Barrett and Russell, 1999). The mood items were averaged together for a daily negative mood score (within-person reliability = 0.54) and a daily positive mood score (within-person reliability = 0.70). Next, participants were asked about stress using a single-item measure validated by Elo et al. (2003). They were instructed: “Stress means a situation in which a person feels tense, restless, nervous, or anxious. Did you feel that kind of stress today?” answered on a 5-point scale [1 (Not at all), 2 (Only a little), 3 (To some extent), 4 (Rather much), or 5 (Very much)].

In the second section of the daily survey, participants were asked to record their food allergy-related issues. They were instructed: “The following questions will relate specifically to problems or issues you may have experienced TODAY as a result of your food allergy.” First, they were asked “Have any food allergy related issues affected you today? See list below as a guide.” [Please refer to **Table 4** for the list of issues and Appendix A Supplementary Materials for wording in the daily survey.] If participants answered “Yes,” they were asked to “select which food allergy related issues affected you today (select all options that apply).” Issues were based on the results of focus group research (Peniamina et al., 2014) which identified in an inductive thematic analysis the most commonly experienced issues people with food allergies face in their daily lives. These issues were thematically grouped into different categories related to allergen-free eating, financial cost, time cost, personal cost, behavior of others, physical effects, psychological issues, and “other.” If participants selected “other” from the list, they were asked to explain briefly what other issue/s they experienced that day. The total number of food issues per day (0–25) was summed within days for each participant.

Note that the questions about daily mood and stress were assessed separately from the assessment of allergy issues. By measuring them separately, we could assess the naturally-occurring relationship between the total number of allergy issues that day and participants' corresponding mood or stress experienced that day, or reactivity (Bolger and Schilling, 1991). With this approach, someone with greater reactivity would feel worse psychologically (higher stress, lower mood) on days they experienced more (vs. fewer) food allergy issues. Although ratings of stress and mood will be influenced by other factors aside from allergy issues, this approach allowed us to quantify the extent to which their overall ratings of stress and mood were yoked to their experiences of allergy issues, and, importantly, whether personality moderated these associations.

### Statistical analysis

Descriptive statistics were computed on the five personality traits and the number of food allergy issues experienced each day (the mean was computed by averaging the number of daily food allergy issues across the 2 weeks; and the maximum was computed by taking the day with the highest number of daily food allergy issues).

Preliminary analyses tested the relationship between personality and *the type of food allergy*. Independent samples *t*-tests were conducted comparing scores on each of the Big Five personality traits between people who did, vs. did not, endorse each food allergen.

For the main analyses, we tested the relationship between personality and *the number of allergy issues*. Correlations were computed between scores on each personality trait (neuroticism, extraversion, openness, agreeableness, conscientiousness) and the mean number of food allergy issues and the maximum number of food allergy issues to understand whether people with different personality traits reported more (or fewer) allergy issues on average or on peak days, respectively. These correlations were adjusted for (i) age because there were age-related differences in four of the five personality traits (correlation between age and neuroticism, *r* = −0.339, *p* < 0.001; extraversion *r* = 0.200, *p* < 0.05; openness *r* = 0.202, *p* < 0.05; agreeableness *r* = 0.117, *p* = 0.229; conscientiousness *r* = 0.262, *p* < 0.01) and (ii) allergy type which was coded with two dummy codes (other foods, gluten/wheat, and peanut/treenut, coded 0, 1, 0, and 0, 0, 1) because people with a gluten/wheat allergy experienced more allergy-related issues than people with other allergy types [without vs. with a gluten/wheat allergy on mean allergy issues per day *M* = 1.5 vs. 2.5 issues/day *t*_(106)_ = −2.428, *p* < 0.05 equal variances not assumed, and max allergy issues per day *M* = 4.9 vs. 6.6 issues/day *t*_(106)_ = −2.208, *p* < 0.05 equal variances assumed].

Next, we tested the relationship between personality and *the likelihood of reporting specific food allergy issues*. Correlations were computed between scores on each personality trait and the likelihood of each of the 25 specific food allergy issues [expressed as a proportion score indicating the proportion of days each food allergy issue was experienced, ranging from 0.000 (no days with that issue) to 1.000 (all days with that issue)].

Lastly, we tested whether personality moderated participants' *psychological reactivity to allergy issues*. We used the Hierarchical Linear Modeling Program (Raudenbush et al., 2011) to model the within-person relationship between the number of food allergy issues experienced each day (level-1 predictor, person-centered) and participants' stress/mood levels that day (level-1 outcome), and how this relationship varied by neuroticism, extraversion, openness, agreeableness, and conscientiousness (Level-2 predictors, all grand-mean centered, entered together). Age of participants (grand-mean centered) and allergy type (other foods, gluten, and peanut/treenut, coded 0, 1, 0, and 0, 0, 1) were entered as additional level-2 control variables. The mulitlevel equations are shown in Appendix B, Supplementary Materials.

## Results

### Participants

A total of 131 participants started the study; 22 participants were excluded from the final sample because they completed fewer than five daily surveys (which we set as a minimum criteria for inclusion because any fewer than this number precluded within-person data analysis), and one participant was excluded because he/she did not fit the criteria for food allergy diagnosis. Socio-demographic, food allergy, and personality characteristics of excluded individuals did not differ from included individuals. The remaining 108 participants (15% male, 85% female) completed at least five daily surveys during the 2-week period (*M* = 10.6 surveys completed, *SD* = 2.5, range 5 to 14). The participants' mean age was 40.2 (range 18 to 87). The sample included participants with a range of food allergies and symptoms (Table 1). Several participants (*n* = 19) reported allergies to three or more foods. The five most common food allergies were to gluten, peanuts, cow's milk, shellfish/seafood, and treenuts.

**Table 1:** Type of food allergy and symptoms of participants.

**Type of food allergy**	**Number of participants/%^a^**	**Symptoms**	**Number of participants/%^a^**
Gluten	59/54.6%	Gastrointestinal	85/78.7%
Peanut	23/21.3%	Skin	58/53.7%
Cow's milk	18/16.7%	Respiratory	32/29.6%
Shellfish/seafood	18/16.7%	Anaphylaxis	26/24.1%
Treenut	17/15.7%	Other^d^	30/27.8%
Egg	14/13.0%		
Fruit/vegetables^b^	12/11.1%		
Wheat	11/10.2%		
Soya	8/7.4%		
Other^c^	10/9.3%		

a * Percentages do not total 100% because participants had multiple food allergies and symptoms*.

b * Fruit/vegetables: avocado (n = 5), kiwifruit (n = 4), banana (n = 3), tomato (n = 2), citrus fruit, lychee, tamarillo, pineapple, capsicum, corn, carrot, spinach*.

c * Other foods: sesame (n = 2), chicken, beef, rice, broad beans, chickpeas, cocoa, peppermint, guar gum, spirulina*.

d * Other symptoms: fatigue, headache/migraine, depression, dizziness, hotness/sweating, blurred vision, drowsiness, anxiety, confusion, lack of concentration, memory loss, edema, oral allergy, mouth ulcers, anemia, weight loss, leg pain, joint problems*.

### Descriptive statistics and preliminary tests

Descriptive statistics for the participants' personality characteristics are given in Table 2. The mean scores for neuroticism, extraversion, and openness fell within the 95% confidence intervals reported by Schmitt et al. (2007) for a population sample from Oceania (Australia, New Zealand, and Pacific Islands; *N* = 926; sample of primarily college students). The mean scores for conscientiousness and agreeableness were higher in comparison with scores reported by Schmitt et al. (2007). The correlations between the personality traits were consistent with prior research showing significant correlations among neuroticism, agreeableness and conscientiousness, a significant correlation between extraversion and openness, and a negative correlation between neuroticism and extraversion (DeYoung, 2015).

**Table 2:** Descriptive statistics and correlations among the personality traits.

	***M***	***SD***	**Min–Max**	**Extra**	**Open**	**Agree**	**Consc**
Neuroticism	2.89	0.80	1.13–5.00	−0.256^**^	0.052	−0.320^**^	−0.346^***^
Extraversion	3.32	0.82	1.50–5.00	1	0.269^**^	0.294^**^	0.199^*^
Openness	3.73	0.65	1.80–5.00		1	0.014	−0.016
Agreeableness	4.00	0.59	2.00–5.00			1	0.321^**^
Conscientiousness	4.02	0.59	2.11–4.89				1

*The minimum score possible for each personality trait was 1 and the maximum score possible was 5. SD = Standard deviation*.

* p < 0.05;

** p < 0.01;

*** *p < 0.001*.

Descriptive statistics for the average number of food allergy issues showed that participants reported experiencing two allergy issues per day [*M* (*SD*) = 2.1 (2.4), range 0–14.4] and six issues per day on peak days [*Max* (*SD*) = 6.0 (4.1), range 0–19.0]. There was substantial range in the number of issues experienced each day. On the low end, seven people reported no allergy issues throughout the entire duration of the 2-week study. On the high end, nine people reported experiencing between five and 14 issues every single day during the study. Similar variation was observed in the maximum number of daily issues.

Descriptive statistics for the specific food allergy issues are presented in Appendix C, Supplementary Materials. As reported in Peniamina et al. (2016), the two most frequently experienced issues were the experience of physical symptoms (experienced on 17.4% of days) and extra financial costs due to higher food prices for safe foods (17.0% of days). This was followed by problems with finding suitable foods to eat when away from home (15.3%), having to take risks by eating foods that may contain allergens (13.4%), and loss of time due to sourcing safe food (12.4%). Again, there was substantial range in the proportion of days issues were experienced. For most issues, some participants never reported experiencing that issue (min = 0.000) and other participants reported experiencing that issue every day surveyed (max = 1.000).

### Personality and the type of allergy

There was no relationship between the Big Five personality traits and the types of foods allergies people had. Independent samples *t*-tests showed no differences in any of the Big Five personality traits between people who did, vs. did not, endorse each food allergen (data available from first author).

### Personality and the number of allergy issues

Table 3 shows the correlations between the number of allergy issues and each of the Big Five personality traits. Openness to experience predicted a higher number of food allergy issues. People one standard deviation above the mean in openness reported the most food allergy issues (2.7 issues per day) and they had the highest maximum number of food allergy issues on a given day (7.3 max issues); by contrast, people one standard deviation below the mean in openness reported the fewest food allergy issues (1.4 issues per day) and they had the lowest maximum number of food allergy issues on a given day (4.6 max issues). Contrary to predictions, there was no association between neuroticism or any other personality trait and the number of food allergy issues.

**Table 3:** Correlations between the mean and maximum number of food allergy issues per day and each personality trait, adjusted for age and allergy type.

	**Neur**	**Extra**	**Open**	**Agree**	**Consc**
Mean issues/day	0.067	0.074	0.268^**^	0.120	−0.097
−1/+1 *SD*	1.9/2.2	1.9/2.2	1.4/2.7	1.8/2.4	2.3/1.8
Max issues/day	0.078	0.085	0.313^**^	0.060	0.002
−1/+1 *SD*	5.6/6.3	5.6/6.3	4.6/7.3	5.7/6.2	5.9/6.0

*r = Pearson correlation coefficient; −1/+1 standard deviation (SD) reflects number of food allergy issues for people low/high in trait. Neur, neuroticism; Extra, extraversion; Open, Openness to experience; Agree, agreeableness; Consc, conscientiousness*.

** *p < 0.01*.

*Bolded numbers are statistically significant*.

### Personality and specific allergy food issues

Table 4 shows the correlations between the likelihood of reporting specific food allergy issues and Big Five personality traits. Contrary to predictions, neuroticism was not associated with a higher frequency of any specific issues. Instead, openness was the strongest predictor of specific issues. People higher in openness were more likely to report issues related to allergen free eating, namely having to go hungry or not eat because there is no safe food available and having problems finding suitable foods when grocery shopping. They also reported significant time costs (having to spend more time preparing food), significant personal and social costs (feeling anxious at social occasions involving food, lack of kindness and understanding from others, being excluded) and psychological issues (feeling embarrassed and inadequate as a result of their food allergy, and feeling anxious about revealing their allergy to others). Extraverted people reported somewhat more issues related to the personal and social challenges of living with a food allergy, such as people being unkind toward them and feeling anxious or stressed in social occasions (trend) and lack of understanding from others (trend), although the patterns were not as strong as for openness. Agreeable people mainly felt anxious or stressed in social occasions involving food. Lastly, conscientiousness had a mixed profile. Although conscientious people had problems finding a restaurant or café, they reported fewer psychological issues (less embarrassment and anxiety about revealing their allergy) than people lower in conscientiousness who reported experiencing more problems.

**Table 4:** Correlations between specific food allergy issues and each personality trait, adjusted for age and allergy type.

	**Neur**	**Extra**	**Open**	**Agree**	**Consc**
**ALLERGEN FREE EATING**					
Having to go hungry/not eat because there is no safe food available.	0.060	0.011	0.264^**^	0.022	−0.141
Problems with finding suitable foods to purchase when grocery shopping.	−0.014	−0.022	0.206^*^	0.143	−0.020
Problems finding a restaurant or café that can provide an allergen free meal.	0.023	0.188^+^	0.127	0.048	0.210^*^
Having to take risks by eating foods that may contain allergens.	0.046	0.054	0.126	0.168^+^	−0.182^+^
Problems with finding suitable foods to eat when away from home (e.g., no safe foods, no healthy options).	−0.045	0.082	0.157	0.073	0.068
Missing out on foods because of the cost of allergen free products.	0.034	0.094	0.156	0.051	−0.084
Trouble with maintaining a healthy, nutritionally balanced diet as a result of my food allergy.	0.096	−0.073	0.149	0.131	−0.052
**FINANCIAL AND TIME COSTS**					
Loss of time due to extra time spent preparing meals.	0.020	−0.027	0.191^*^	0.047	−0.069
Extra financial cost due to medical expenses resulting from the food allergy (doctor's visits regarding food allergy, treatment for food allergy symptoms).	−0.005	0.022	0.171^+^	0.088	−0.005
Loss of time due to extra time spent sourcing safe food (e.g., reading labels, going to different shops).	−0.017	−0.012	0.156	0.051	0.087
Extra financial cost due to higher food prices for safe foods.	0.012	0.040	0.145	0.091	−0.007
Loss of time due to extra time spent organizing food (e.g., packing safe food to bring along when out, pre-preparing meals to store in freezer).	0.003	−0.043	0.009	−0.078	0.017
**PERSONAL AND SOCIAL COSTS**					
Feeling anxious or stressed when participating in social occasions involving food.	0.134	0.188^+^	0.340^***^	0.219^*^	−0.127
People being uncooperative or unkind toward me because of my food allergy.	0.023	0.241^*^	0.245^*^	0.161^+^	−0.064
Lack of understanding from others in relation to my food allergy.	0.097	0.164^+^	0.288^**^	0.145	−0.110
Being excluded from social occasions because of food allergy.	0.134	0.083	0.257^**^	0.057	−0.066
Avoiding participation in social occasions because of food allergy.	0.057	0.092	0.168^+^	−0.040	−0.073
Difficulties traveling with food allergies.	−0.026	0.058	0.167^+^	−0.037	0.033
**PHYSICAL EFFECTS**					
Physical symptoms of food allergy.	0.079	−0.012	0.092	0.022	−0.130
**PSYCHOLOGICAL ISSUES**					
Feeling embarrassed as a result of my food allergy.	0.114	0.116	0.307^**^	0.130	−0.202^*^
Feeling inadequate or defective as a result of my food allergy.	0.073	0.157	0.247^*^	0.198^*^	−0.119
Feeling anxious about how people will react if I reveal my food allergy.	0.092	0.096	0.228^*^	0.119	−0.199^*^
Feeling anxious about whether food is safe to eat.	0.074	−0.020	0.176^+^	0.132	−0.139
Feeling anxious about potentially having an allergic reaction.	0.041	−0.021	0.138	0.018	−0.092
Other issues	−0.015	0.036	0.075	0.031	−0.004

*Neur, neuroticism; Extra, extraversion; Open, Openness to experience; Agree, agreeableness; Consc, conscientiousness*.

+ p < 0.10;

* p < 0.05;

** p < 0.01;

*** *p < 0.001*.

*Bolded numbers are statistically significant*.

### Personality and psychological reactivity to allergy issues

Table 5 summarizes the results of the multilevel models and how each Big Five personality trait moderated the within-person relationship between the number of daily food allergy issues and experiences of stress, negative mood, and positive mood, controlling for age and allergy type. Significant moderation effects were found for extraversion, which had a buffering effect on stress and negative mood reactivity. People higher in extraversion experienced less of an increase in stress and negative mood on days with more food allergy issues, compared to people lower in extraversion. A significant moderation effect was also found for conscientiousness. Conscientiousness exacerbated the negative impact of food allergy issues on positive mood. People higher in conscientiousness experienced a greater reduction in positive mood on days with more allergy issues, compared to people lower in conscientiousness.

**Table 5:** How personality traits moderated the within-person relationship between daily food allergy issues and stress, negative mood, and positive mood, adjusting for age and allergy type.

**Predictors**		**Stress *B* (*SE*)**	**Negative mood *B* (*SE*)**	**Positive mood *B* (*SE*)**
Intercept (*G*_00_)	G_00_	0.939 (0.101)^***^	1.702 (0.067)^***^	3.061 (0.109)^***^
Age	G_01_	−0.011 (0.004)^**^	−0.012 (0.002)^***^	−0.004 (0.004)
Allergy Type Dummy1	G_02_	0.152 (0.119)	−0.023 (0.076)	0.116 (0.118)
Allergy Type Dummy2	G_03_	−0.182 (0.140)	0.024 (0.095)	0.019 (0.119)
Neuroticism	G_04_	0.131 (0.068)^+^	0.149 (0.055)^**^	−0.204 (0.056)^***^
Extraversion	G_05_	0.114 (0.068)^+^	0.043 (0.048)	0.260 (0.060)^***^
Openness	G_06_	0.114 (0.087)	0.071 (0.059)	0.043 (0.079)
Agreeableness	G_07_	0.089 (0.106)	0.055 (0.063)	0.051 (0.080)
Conscientiousness	G_08_	−0.143 (0.091)	−0.116 (0.067)^+^	0.216 (0.083)^*^
Issues Slope (*G*_10_)	G_10_	0.096 (0.024)^***^	0.038 (0.015)^*^	−0.018 (0.020)
Age	G_11_	−0.001 (0.001)	0.001 (0.001)	−0.002 (0.001)^**^
Allergy Type Dummy1	G_12_	−0.040 (0.027)	−0.021 (0.017)	0.034 (0.022)
Allergy Type Dummy2	G_13_	0.007 (0.031)	0.017 (0.022)	−0.010 (0.022)
Neuroticism	G_14_	−0.010 (0.020)	−0.006 (0.011)	−0.016 (0.016)
Extraversion	G_15_	−0.043 (0.015)^**^	−0.021 (0.011)^*^	0.012 (0.013)
Openness	G_16_	0.020 (0.021)	−0.005 (0.013)	0.030 (0.020)
Agreeableness	G_17_	0.039 (0.025)	0.022 (0.014)	−0.007 (0.017)
Conscientiousness	G_18_	−0.016 (0.027)	0.015 (0.018)	−0.060 (0.019)^**^

*Coefficients (with robust standard errors) from multilevel models are presented. Coefficient G_10_ is the average reactivity pattern [the within-person relationship between the number of food allergy issues (as a group-centered predictor) and stress, negative mood, or positive mood (as separate outcome variables) for participants of average age without a gluten/wheat allergy or nut allergy and at mean levels on the personality traits]. Coefficients G_14_ to G_18_ reflected how each personality trait uniquely moderated the average reactivity pattern*.

+ p < 0.10;

* p < 0.05;

** p < 0.01;

*** *p < 0.001*.

## Discussion

Our findings suggest that personality plays an important role in how people live and cope with food allergies in their daily life. Surprisingly, neuroticism was not associated with more allergy issues or greater reactivity to allergy issues in our study. This finding contrasts with previous research showing that neuroticism is associated with more symptom over-reporting in general (Larsen, 1992), greater stress reactivity (Bolger and Schilling, 1991; Suls and Martin, 2005), and poorer illness adaptation among people coping with other chronic conditions (Van De Ven and Engels, 2011; Rassart et al., 2013, 2014; Muscatello et al., 2016; Topp et al., 2016).

Instead, openness to experience was associated with the most allergy issues in our study. Openness to experience describes a tendency to seek out broader, deeper, more original, and more complex experiences (John et al., 2008; DeYoung, 2010). It was therefore not surprising that participants with a higher openness score tended to report more allergy issues, as they were likely to be out experiencing more situations that would expose them to those issues. It is also possible that the demands of coping with a food allergy—requiring caution, routine, and consumption of known foods—might be in direct conflict with the open personality that craves stimulation, variation, and novel experiences (DeYoung, 2014). This was evident in some of the specific issues that more open participants reported. For example, the fact that open people had problems finding suitable foods when grocery shopping suggests that their desires for food variety is quite challenging. Also, not eating because there was no safe food available suggests that they might have been doing something unplanned or outside their routine and had not considered the implications for their food allergy or they prioritized other experiences. Open people also reported more personal, social, and psychological issues with their food allergy including being excluded from social occasions, feeling anxious or stressed at social occasions involving food, people being uncooperative or unkind, lack of understanding from others, and greater feelings of embarrassment, inadequacy, and anxiety about revealing their food allergy. However, openness was not associated with greater stress or mood reactivity to allergy issues. So, although highly open people experienced more issues, their mood and level of stress experienced in response to those issues was proportionally similar to other personality types.

The other three traits played relatively less important roles in coping with food allergies.

Extraversion was associated with higher reporting of some personal and social allergy issues (people being unkind), but also conferred benefit in terms of reduced reactivity. According to John et al. (2008) extraverts are social, active, assertive, and possess a positive emotionality. It therefore makes sense that extraverted individuals, being naturally more social and socializing more often would also increase their chances of exposure to people who might be uncooperative or unkind, compared to those who spend less time with others. However, while they reported experiencing these two issues more often, there was no overall increase in number of issues per day associated with extraversion, and this personality trait was associated with lower stress and negative mood reactivity to issues. Their lower negative emotional reactivity is likely to be related to their positive approach to life and their tendency toward more adaptive coping skills (Masthoff et al., 2007; Van De Ven and Engels, 2011). In addition, extraverts are more likely to receive social support (Caspi et al., 2005).

Agreeableness was a minor player. It did not predict the total number of reported food allergy issues nor did agreeableness moderate psychological reactivity to allergy issues. However, participants who were more agreeable were more likely to report feeling anxious or stressed at social occasions involving food. This may be because participants with this socially conscious personality type did not want to upset anyone and therefore felt more anxious and/or stressed about having to assert their needs in a social setting. Not wanting to be bothersome and finding it difficult to be assertive were themes discussed by food allergic participants in previous research (Peniamina et al., 2014). More agreeable people were also more likely to report feeling “inadequate or defective” as a result of their food allergy. This contrasts with the findings of Van De Ven and Engels (2011) and Rassart et al. (2013) who found that higher agreeableness predicted better quality of life in individuals with chronic conditions, due to their tendency to use positive reappraisal. This difference could be explained by the lower perceived social acceptance associated with food allergies in comparison with other chronic conditions such as asthma and congenital heart disease (Peniamina et al., 2014). Thus, agreeable individuals with food allergies may be more likely to hide their condition and expect negative social repercussions if they assert their needs, resulting in lower social support and increased feelings of inadequacy.

Conscientiousness played a mixed role in the day-to-day lives of people with food allergies. The logical rule-following approach of conscientious individuals (Carver and Connor-Smith, 2010) could explain why they were less likely to feel embarrassed because of their food allergy (i.e., they feel justified in their behavior because they are adhering to the rules associated with allergy management). Conversely, their desire to follow the rules and avoid risk-taking may also explain why they were more likely to report problems finding a restaurant or café that could provide an allergen-free meal. Further, conscientious people were more reactive to allergy issues, showing greater decrements in positive mood on days with more allergy issues compared to their less conscientious peers. This finding runs counter to previous research showing a protective role of higher conscientiousness in coping with other chronic conditions (Rassart et al., 2014; Muscatello et al., 2016; Topp et al., 2016) and research by Gartland et al. (2014) who found that more conscientious people were *less* reactive to daily hassles (i.e., they had a smaller decrease in positive affect on days with more hassles). We are not sure what may be causing this difference except that conscientious people may have felt that the occurrence of allergy issues indicated a failure on their part–issues that could have been avoided with better planning.

There were several strengths and limitations of our study. Strengths included our daily diary design, representation of a wide range of personalities in the study, and requirement that participants had to have a physician-diagnosed food allergy rather than a self-assessed allergy. However, this strict criterion made it difficult to recruit a larger number of participants. Although a relatively small sample size of 108 participants may be viewed as a weakness, the sample size is comparable to other diary studies of people with chronic conditions (e.g., *n* = 54 with rheumatoid arthritis; Affleck et al., 1992; *n* = 70 with chronic pain; Rost et al., 2016) and should be considered in light of the intensive nature of data collection and the multilevel design. Furthermore, a study examining the issue of appropriate sample size in multilevel modeling found that issues only occurred with a level-2 sample size (N) of 50 or fewer (Maas and Hox, 2005). Although participants in our study expressed a full range of personality traits, the mean scores for conscientiousness and agreeableness were higher in comparison with normative scores for an Oceania sample reported by Schmitt et al. (2007). This difference is likely to be related to the age of our participants because both conscientiousness and agreeableness have been shown to increase with age (Srivastava et al., 2003). While age statistics were not reported by Schmitt and colleagues, their study sample consisted primarily of college students while our study included participants aged between 18 and 87.

Although we assessed a large number of food allergy issues derived inductively from a previous qualitative study (Peniamina et al., 2014), this list may be incomplete. There could be other food allergy issues that participants from our previous qualitative study did not raise (Peniamina et al., 2014). Also, although we analyzed the food allergy issues separately in order to maintain granularity in findings, the categories of issues may benefit from further refinement and psychometric testing. Currently, the issues are grouped together on the basis of face similarity, derived inductively from qualitative analysis; however, there may be other ways to arrange the issues instead of the categories we used. Exploratory factor analysis could be used to regroup the list into a consolidated measure of food allergy issues.

It is also difficult to say whether the prevalence of food allergies found in this study reflects the prevalence in the population of other adults with food allergies. Most of the prior research reports the percentage of people with various food allergies in the entire population, not just the estimates of different food allergies in adults with food allergies. However, the approximate 50:50 split we found between those with coeliac disease (gluten) and other allergies combined is likely an accurate representation. Also, our data are similar to a meta-analysis which found food allergies in the following rank order (excluding gluten): cow's milk 0.6%, tree nuts 0.5%, soy 0.3%, peanuts 0.2%, egg 0.2%, wheat 0.1%, fish 0.1%, and shellfish 0.1% (Nwaru et al., 2014). Our ranking was similar, but not identical: peanuts, cow's milk, shellfish/seafood, treenuts, egg, fruit/vegetables, wheat, and soy. Peanut allergies may have been overrepresented in our study sample. Since peanut allergies are often severe allergies, which might be more likely to be medically diagnosed, a higher proportion of people with peanut allergies would be evident among people recruited with a medically-diagnosed food allergy vs. people recruited using other criteria or from a random sample of the general population.

There are several implications and future directions of this work. Based on our results, clinicians should be able to predict which patients are having difficulty adapting to life with food allergies and why that might be the case. We suggest that people high in openness will be at greatest psychological risk because their personalities are in direct conflict with some of the behavioral requirements of living with a food allergy (which requires greater caution, safety focus, and routine). Our results may also help people with food allergies to understand how their own personality might affect their food allergy experiences, and how they could adopt strategies to improve coping with their food allergies. For example, open people could try to satisfy their novelty needs on other experiences aside from food and they could bring food with them to allow for spontaneous changes in situations. Extraverted people could plan social events in their own homes where they have more control over food. Agreeable people could practice being more disagreeable by speaking up to others about their food allergy, which should become easier over time. And, more conscientious people could try to be kinder to themselves when food allergy issues arise in their daily lives.

The present findings also suggest that openness and the other personality traits should be examined in children with food allergies. Research suggests that food allergies affect 4–8% of children (Sicherer, 2011; 7%, Hill et al., 2016) and can present numerous challenges to the children and parents (Primeau et al., 2000; Sicherer et al., 2001; Bollinger et al., 2006; King et al., 2009; LeBovidge et al., 2009). Understanding a child's personality disposition, such as to openness, could help parents enact strategies to work with, rather than against, their child's personality to help them manage their food allergy.

## Conclusion

Personality traits matter in the experiences of adults with food allergies. Although we predicted that people higher in neuroticism would have more problems managing the day-to-day experiences of living with a food allergy, this hypothesis was not supported. Instead, the data showed that people higher in trait openness had the greatest difficulty managing their food allergies in daily life. The types of issues open people faced included problems with allergy free eating, social circumstances (how to navigate social occasions involving food, lack of understanding from others) and greater psychological issues (feeling embarrassed and inadequate). Such difficulties shouldn't be seen as a failure on their part, but rather a conflict between the constraints of living with food allergies and the core features of openness. Strategies to manage food allergies and improve quality of life in both adults and children will be more effective by considering core differences in personality.

## Ethics statement

This study was carried out in accordance with the recommendations of the Ethical Practices in Research Involving Human Participants from the University of Otago Ethics Committee with electronic informed consent from all subjects. All subjects gave electronic informed consent in accordance with the Declaration of Helsinki. The protocol was approved by the Departments of Psychology and Food Science at the University of Otago, New Zealand.

## Author contributions

TC and RP conceived the idea, planned the study, conducted data analysis, and co-wrote the manuscript. TC provided additional supervisory support to RP. MM conceived the idea, contributed to the manuscript, and provided ongoing supervisory support to RP. PB contributed to the manuscript and provided ongoing departmental support to RP. All authors gave their approval for the final manuscript.

### Conflict of interest statement

The authors declare that the research was conducted in the absence of any commercial or financial relationships that could be construed as a potential conflict of interest.
