# Supplementary material for: The Role of Personality in Daily Food Allergy Experiences
Source: Front Psychol. 2018 Feb 6;9:29. doi: 10.3389/fpsyg.2018.00029 (PMC5807900; doi:10.3389/fpsyg.2018.00029)
Supplement: Supplementary file 1 [file DataSheet1.pdf]

Supplementary Materials for Conner, Mirosa, Bremer, and Peniamina (2018)

Appendix A. Allergy issues as worded in the daily survey

Appendix B. Multilevel modeling equations

Appendix C. Descriptive statistics for the 25 food allergy issues

Appendix A. Allergy issues as worded in the daily survey

The following questions will relate specifically to problems or issues you may have experienced TODAY as a result of your food allergy.

1. Have any food allergy related issues affected you today? (see list below as a guide)
   ☐ YES ☐ NO

If participants selected YES they were asked to complete following question:

2. Please select which food allergy related issues affected you today (select all options that apply):

Allergen free eating

☐ Problems with finding suitable foods to eat when away from home (e.g., no safe foods, no healthy options).
☐ Problems with finding suitable foods to purchase when grocery shopping.
☐ Having to take risks by eating foods that may contain allergens.
☐ Having to go hungry/not eat because there is no safe food available.
☐ Problems finding a restaurant or café that can provide an allergen free meal.
☐ Missing out on foods because of the cost of allergen free products.
☐ Trouble with maintaining a healthy, nutritionally balanced diet as a result of my food allergy.

Financial cost

☐ Extra financial cost due to higher food prices for safe foods.
☐ Extra financial cost due to medical expenses resulting from the food allergy (e.g., doctor’s visits regarding food allergy, treatment for food allergy symptoms).

Time cost

☐ Loss of time due to extra time spent sourcing safe food (e.g., reading labels, going to different shops).
☐ Loss of time due to extra time spent preparing meals.
☐ Loss of time due to extra time spent organizing food (e.g., packing safe food to bring along when out, pre-preparing meals to store in freezer).

Personal cost

☐ Avoiding participation in social occasions because of food allergy.
☐ Being excluded from social occasions because of food allergy.
☐ Feeling anxious or stressed when participating in social occasions involving food.
☐ Difficulties travelling with food allergies.

External influences

☐ Lack of understanding from others in relation to my food allergy. Select the situation/s in which this was experienced today:
- ☐ At the doctor
- ☐ Dealing with other medical professionals
- ☐ At a restaurant/café (involving restaurant/café staff)
- ☐ At a social gathering (dealing with peers)
- ☐ At a family gathering (dealing with family members)
- ☐ At work
- ☐ Other, please describe briefly:

☐ People being uncooperative or unkind towards me because of my food allergy. Select the situation in which this was experienced today:
- ☐ At the doctor
- ☐ Dealing with other medical professionals
- ☐ At a restaurant/café (involving restaurant/café staff)
- ☐ At a social gathering (dealing with peers)
- ☐ At a family gathering (dealing with family members)
- ☐ At work
- ☐ Other, please describe briefly:

Physical effects

☐ Physical symptoms of food allergy. What type of symptoms did you experience today? (Please select all options that apply)
- ☐ Cardiovascular symptoms (e.g., lightheadedness, dizziness, palpitations, loss of vision, collapse, loss of consciousness)
- ☐ Respiratory (e.g., shortness of breath, difficulty breathing, wheezing)
- ☐ Gastrointestinal (e.g., nausea, abdominal cramps, vomiting, diarrhoea)
- ☐ Skin (e.g., itchy skin, red rash, eczema, hives, swelling of the skin)
- ☐ Nose/eyes (e.g., itchy eyes, watery eyes, nasal congestion, sneezing)
- ☐ Oral (swelling or itchiness of mouth/tongue/throat)
- ☐ Other, please list:

Psychological issues

☐ Feeling anxious about whether food is safe to eat.
☐ Feeling anxious about potentially having an allergic reaction.
☐ Feeling embarrassed as a result of my food allergy.
☐ Feeling inadequate or defective as a result of my food allergy.
☐ Feeling anxious about how people will react if I reveal my food allergy.

Other issues

☐ Other, please describe briefly:

Appendix B. Multilevel modeling equations

We ran three multilevel models using the Hierachical Linear Modeling Program (version 6.04; Raudenbush, Bryk, & Congdon, 2011). One model was run for each outcome (stress, negative mood, positive mood). The level-1 outcome (daily stress, negative mood, or positive mood) was predicted by the number of food allergy issues experienced that day (Issues), plus age and allergy type as covariates and personality traits as level-2 predictors. The example equations for stress are as follows:

Level-1 Equation

$$\text{Stress} = B_0 + B_1 * (\text{Issues}) + r$$

Level-2 Equations

$$B_0 = G_{00} + G_{01}*(\text{Age}) + G_{02}*(\text{Gluten}) + G_{03}*(\text{Nut}) + G_{04}*(\text{Neur}) + G_{05}*(\text{Extr}) + G_{06}*(\text{Open}) + G_{07}*(\text{Agre}) + G_{08}*(\text{Cons}) + U_0$$

$$B_1 = G_{10} + G_{11}*(\text{Age}) + G_{12}*(\text{Gluten}) + G_{13}*(\text{Nut}) + G_{14}*(\text{Neur}) + G_{15}*(\text{Extr}) + G_{16}*(\text{Open}) + G_{17}*(\text{Agre}) + G_{18}*(\text{Cons}) + U_1$$

In the level-1 equation, each participant's outcome (stress) was predicted by their average stress (B_0) plus the number of food allergy issues experienced that day (B_1) which was group-mean centred (around each individual's mean) so that B_1 indicated how stress varied on days when a person experienced more (versus fewer) than their average number of allergy issues. This level-1 model yielded an intercept B_0 and slope B_1 for each participant.

In the level-2 equations, participants' intercepts B_0 and slopes B_1 were predicted by age and allergy type as covariates and the personality traits of neuroticism, extraversion, openness, agreeableness, and conscientiousness, as simultaneous predictors[1]. Continuous variables were grand-mean centered. Gluten was coded 0 or 1 for absence or presence of a gluten/wheat allergy. Nut was coded 0 or 1 for absence or presence of a peanut/treenut allergy. The level-2 grand intercept (G_{00}) reflected the average stress for participants at the average sample age who did not endorse either a gluten, wheat, peanut or treenut allergy, and who scored average on the personality traits. Coefficients G_{01} - G_{08} tested whether age, allergy type, or personality traits predicted differences in average stress. The level-2 grand slope (G_{10}) reflected the average reactivity patterns for the sample, i.e. the within-person relationship between daily food allergy issues and stress for participants at the average sample age, without a gluten, wheat, peanut, or treenut allergy, and who scored average on the personality traits. Coefficients G_{11} - G_{18} tested whether age, allergy type, or personality traits predicted differences in these reactivity patterns. For example, a significant G_{14} coefficient would indicate that the within-person relationship between allergy issues and stress varied as a function of neuroticism, controlling for age and allergy type.

Table 5 presents the results of these models in the form of the Level-2 intercept coefficients G_{00} - G_{08} (top) and Level-2 slope coefficients G_{10} – G_{18} (bottom).

Footnote

[1] Models testing each personality trait separately showed nearly identical patterns of results.

Appendix C. Descriptive statistics for the 25 food allergy issues expressed. Numbers are proportion scores, reflecting the proportion of days each issue was experienced by participants.

	Mean	*SD*	*Min*	*Max*
**Allergen free eating**				
Having to go hungry/not eat because there is no safe food available.	0.099	0.151	0.000	0.750
Problems with finding suitable foods to purchase when grocery shopping.	0.060	0.094	0.000	0.500
Problems finding a restaurant or café that can provide an allergen free meal.	0.046	0.068	0.000	0.230
Having to take risks by eating foods that may contain allergens.	0.134	0.185	0.000	1.000
Problems with finding suitable foods to eat when away from home (e.g., no safe foods, no healthy options).	0.153	0.180	0.000	0.830
Missing out on foods because of the cost of allergen free products.	0.057	0.164	0.000	1.000
Trouble with maintaining a healthy, nutritionally balanced diet as a result of my food allergy.	0.112	0.208	0.000	1.000
**Financial and Time Costs**				
Loss of time due to extra time spent preparing meals.	0.102	0.186	0.000	0.890
Extra financial cost due to medical expenses resulting from the food allergy (doctor's visits regarding food allergy, treatment for food allergy symptoms).	0.031	0.118	0.000	1.000
Loss of time due to extra time spent sourcing safe food (e.g., reading labels, going to different shops).	0.124	0.179	0.000	1.000
Extra financial cost due to higher food prices for safe foods.	0.170	0.229	0.000	1.000
Loss of time due to extra time spent organizing food (e.g., packing safe food to bring along when out, pre-preparing meals to store in freezer).	0.085	0.166	0.000	1.000
**Personal and social costs**				
Feeling anxious or stressed when participating in social occasions involving food.	0.085	0.143	0.000	0.830
People being uncooperative or unkind towards me because of my food allergy	0.015	0.067	0.000	0.500
Lack of understanding from others in relation to my food allergy.	0.082	0.159	0.000	1.000
Being excluded from social occasions because of food allergy.	0.035	0.093	0.000	0.600
Avoiding participation in social occasions because of food allergy.	0.065	0.108	0.000	0.600
Difficulties travelling with food allergies.	0.041	0.110	0.000	0.630
**Physical effects**				
Physical symptoms of food allergy.	0.174	0.253	0.000	1.000

	Mean	*SD*	*Min*	*Max*
**Psychological issues**				
Feeling embarrassed as a result of my food allergy.	0.076	0.135	0.000	0.830
Feeling inadequate or defective as a result of my food allergy.	0.047	0.127	0.000	1.000
Feeling anxious about how people will react if I reveal my food allergy.	0.031	0.087	0.000	0.670
Feeling anxious about whether food is safe to eat.	0.115	0.164	0.000	1.000
Feeling anxious about potentially having an allergic reaction.	0.081	0.144	0.000	0.830
**Other issues**	0.049	0.106	0.000	0.630

Note. The mean proportion score is the average proportion of days that issue was reported by participants in the daily diaries; the standard deviation (*SD*) reflects the degree of variation between participants in the proportion scores; the minimum (min) is the lowest proportion score observed (typically 0.000 indicating that at least one participant reported never experiencing that issue); the maximum (max) is the highest proportion score observed (typically 1.000 indicating that at least one participant reported experiencing that issue every single day surveyed). Numbers can be converted into percentage scores by multiplying by 100.
